# Leishmaniasis transmission in an ecotourism area: potential vectors in Ilha Grande, Rio de Janeiro State, Brazil

**DOI:** 10.1186/1756-3305-6-325

**Published:** 2013-11-13

**Authors:** Bruno Moreira Carvalho, Michele Maximo, Wagner Alexandre Costa, Antonio Luís Ferreira de Santana, Simone Miranda da Costa, Taiana Amancio Neves da Costa Rego, Daniela de Pita Pereira, Elizabeth Ferreira Rangel

**Affiliations:** 1 Laboratório de Transmissores de Leishmanioses, Instituto Oswaldo Cruz, Fundação Oswaldo Cruz. Av. Brasil, 4365, Pavilhão Carlos Chagas, 5° andar, sala 43 – Manguinhos, Rio de Janeiro, RJ 21040-360, Brasil; 2 Fundação Municipal de Saúde de Angra dos Reis, Prefeitura Municipal de Angra dos Reis. Praça General Osório 36, Centro, Angra dos Reis, RJ 23900-600, Brasil; 3 Laboratório de Biologia Molecular e Doenças Endêmicas, Instituto Oswaldo Cruz, Fundação Oswaldo Cruz. Av. Brasil, 4365, Pavilhão Leônidas Deane, sala 209 – Manguinhos, Rio de Janeiro, RJ 21040-360, Brasil

**Keywords:** Sand fly vectors, Touristic area, Visceral leishmaniasis, Cutaneous leishmaniasis, Rio de Janeiro

## Abstract

**Background:**

The south coast of Rio de Janeiro State, in Brazil, is endemic for cutaneous and visceral leishmaniases and is frequently visited by tourists from different parts of the world. Since the complex epidemiology of leishmaniases demands local studies, the goal of this study was to investigate the phlebotomine sand fly fauna and leishmaniases transmission in Ilha Grande, an ecotourism area of Angra dos Reis municipality.

**Methods:**

Sand fly fauna was sampled in three monitoring stations using HP light traps in domiciles, peridomiciles and forests. Species abundance was evaluated by the Index of Species Abundance. A *Leishmania* natural infection survey was done using multiplex PCR and dot blot hybridization.

**Results:**

During 15 consecutive months of sand fly monitoring, 1093 specimens from 16 species were captured. The potential leishmaniases vectors found were *Lutzomyia (Nyssomyia) intermedia*, *L. migonei*, *L. (N.) flaviscutellata, L. (Psychodopygus) ayrozai* and *L. (Lutzomyia) longipalpis*. Five species were new records in Ilha Grande: *L. (Sciopemyia) microps*, *L. termitophila*, *L. firmatoi*, *L. rupicola* and *L. (P.) ayrozai*. Higher species richness was found inside forest areas, although potential leishmaniases vectors were present in deforested areas, peridomiciles and inside houses. *Lutzomyia (N.) intermedia* and *L. migonei* were the most abundant species. Females of *L. migonei* showed a high rate (10.3%) of natural infection by *Leishmania (Viannia)* sp., probably *Leishmania (V.) braziliensis*.

**Conclusions:**

The detection of leishmaniases transmission and potential vectors in Ilha Grande is of public health concern, especially because tourists are frequently visiting the island. Besides reinforcing the epidemiological importance of *L. (N.) intermedia* in Rio de Janeiro State, the role of *L. migonei* in cutaneous leishmaniasis transmission is highlighted with its high rate of *Leishmania* natural infection. The finding of *L. (L.) longipalpis* confirmed the human autochthonous case of visceral leishmaniasis from the island. The presence of *L. (N.) flaviscutellata* in peridomestic areas is also an important finding, since the species is involved in the transmission of diffuse cutaneous leishmaniasis. Health education practices directed to the local community and tourists are important control actions that can be taken in Ilha Grande to reduce the burden of leishmaniases.

## Background

Phlebotomine sand flies are insects (Diptera: Psychodidae) known for their role in leishmaniases transmission to man, as well as bartonellosis and numerous arboviruses. In the Neotropical region, approximately 500 species are described, with about 20 of them related to leishmaniases transmission [1,2].

In endemic regions, leishmaniases show a diffuse distribution, composed by smaller, local transmission foci. The diversity of vector, parasite and host species involved in the transmission cycles contribute to the complex epidemiology of these diseases. Therefore, environmental changes caused by human or natural events affect local populations of these species and can influence disease risk [3,4].

Leishmaniases are being considered as emerging diseases in travelers. In a study of imported human cases in non-endemic countries, South America was considered the main area for the acquisition of cutaneous leishmaniasis, with adventure travelers on long-term trips to forested areas at higher risk of infection [5].

In Rio de Janeiro State, Brazil, the majority of cutaneous leishmaniasis (CL) human cases are caused by *Leishmania (Viannia) braziliensis*. The most abundant sand fly species in the state’s transmission foci is *Lutzomyia (Nyssomyia) intermedia*, but other potential vector species are also present: *L. migonei*, *L. (N.) whitmani*, *L. (Pintomyia) fischeri* and *L. (N.) flaviscutellata*. Human cases of visceral leishmaniasis (VL) are also reported in the state, which are caused by *Leishmania (Leishmania) infantum chagasi*, and transmitted by its main vector, *Lutzomyia (Lutzomyia) longipalpis *[6,7].

The south coast of Rio de Janeiro State is a very attractive place for tourists seeking the natural beauties of the Atlantic Forest, but it is also the second region of the state most affected by CL, after the metropolitan region. A recent study demonstrated that Angra dos Reis, a municipality in the state’s south coast, is one of the most vulnerable to impacts on diseases caused by climate and environmental change, including CL [8].

In Angra dos Reis municipality the main destination of tourists is Ilha Grande (*“*Big Island*”*). This island has records of sporadic CL human cases since the first outbreak, which occurred in the small fisherman community of Praia Vermelha in 1975 [9,10]. The first sand fly survey of the island was published by Araújo Filho et al. [11,12], who detected potential CL vector species, such as *L. (N.) intermedia*, *L. migonei*, and *L. (N.) flaviscutellata*. The other captured species, without known medical importance, were *Brumptomyia cunhai*, *B. nitzulescui*, *L. edwardsi*, *L. lanei*, *L. pascalei* and *L. (Micropygomyia) schreiberi*. The authors also detected *L. (L.) longipalpis*, but at the time no human case of VL had been recorded on the island.

In 2005, the first and only VL human case of Ilha Grande was recorded in a fisherman’s village at Enseada das Estrelas, approximately 20 km away of Praia Vermelha, where *L. (L.) longipalpis* had been detected in the late 1970s by Araújo Filho & Sherlock [13]. Following the notification of this VL case, the Health Department of Angra dos Reis Municipality (FuSAR) performed sand fly surveys on the area, as part of the entomologic surveillance activities. The surveys detected only CL vector species: *L. (N.) intermedia*, *L. migonei* and *L. (P.) fischeri*. As the VL main vector *L. (L.) longipalpis* was not found, the case was left opened in the Ministry of Health’s Disease Notification System (SINAN). Besides the cited species, FuSAR also has records of *L. (N.) whitmani* and *L. (P.) pessoai* occurrence from other parts of the island [14].

Another sand fly survey conducted in Ilha Grande was a study by Souza et al. [15], who detected *L. (N.) intermedia*, *L. migonei*, *L. tupynambai*, *L. pelloni*, *L. (M.) schreiberi* and *Brumptomyia* sp. in Vila do Abraão. Caldellas [16] performed a canine serological survey on the island and also captured *L. (N.) intermedia* and *L. migonei* in peridomestic areas of Enseada das Estrelas and Vila do Abraão.

Since Ilha Grande is an ecotourism area that receives travelers from many parts of the world, it is important to study the local transmission of infectious diseases. Therefore, the goal of this study was to investigate the phlebotomine sand fly fauna and leishmaniases transmission in the island, which has records of human cases of both CL and VL.

## Methods

### Study area

**Figure 1:**
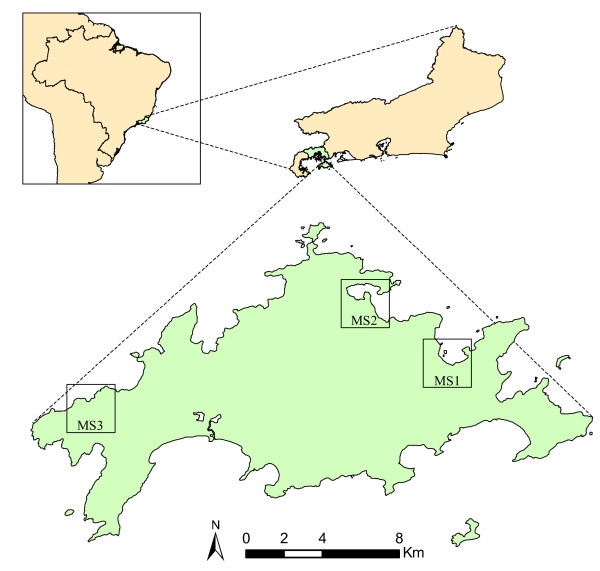
**Location of Ilha Grande in Brazil and Rio de Janeiro State.** Sand fly monitoring stations are highlighted as MS1: Vila do Abraão, MS2: Enseada das Estrelas and MS3: Praia Vermelha.

Ilha Grande is the greatest continental island of Rio de Janeiro State, with area of approximately 193 km^2^. It is located between coordinates 23°5’ – 23°14’ south and 44°51 – 44°23’ west, in the Atlantic Forest biome, in Angra dos Reis municipality (Figure 1). The island is mainly covered by dense ombrophilous forest, and also in smaller proportions, secondary forests, sandy ridges, mangroves and herbaceous vegetation on rocky outcrops. The weather is typically tropical, with higher temperature and precipitation during summer. The annual precipitation is high (over 2,000 mm), with frequent extreme rain events [17]. A few months before the start of this study, in January 2010, a great natural disaster struck the island, when huge landslides occurred in several localities. One of the studied localities was directly affected by these landslides, the community of Praia Vermelha.

The island has a population of 9,000 residents, spread in small villages near beaches. The Atlantic Forest cover is in different regeneration levels due to human occupation and use in the past for agriculture through crops of sugar cane, coffee and corn [18]. Four Environmental Preservation Areas exist on the island to protect the natural ecosystems and have restrictive rules for land use, so touristic activities are considered ecotourism. In fact, ecotourism is the main economic activity of the island, which attracts people from different parts of the world [19].

### Sand fly survey

Three monitoring stations were established to sample the sand fly fauna monthly: Vila do Abraão (MS1), Enseada das Estrelas (MS2) and Praia Vermelha (MS3) (Figure 1). Sporadic CL human cases are recorded in the three localities. Enseada das Estrelas is the only locality of the island with a recorded VL human case. *Leishmania* infections were also detected in dogs in the three localities [11,15,16], as well as in sylvatic and synanthropic rodents in Praia Vermelha [20].

**Figure 2:**
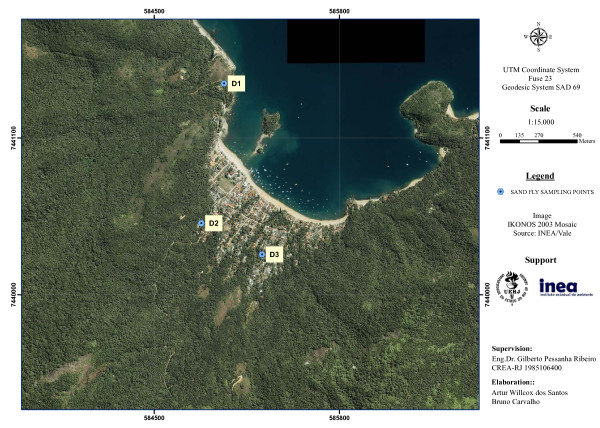
**Sand fly monitoring station 1, Vila do Abraão, showing sampled domiciles.** Ilha Grande, Angra dos Reis (RJ).

**Figure 3:**
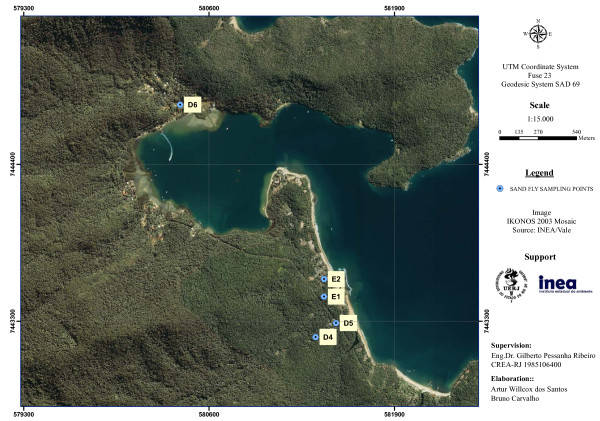
**Sand fly monitoring station 2, Enseada das Estrelas, showing sampled domiciles.** Ilha Grande, Angra dos Reis (RJ).

**Figure 4:**
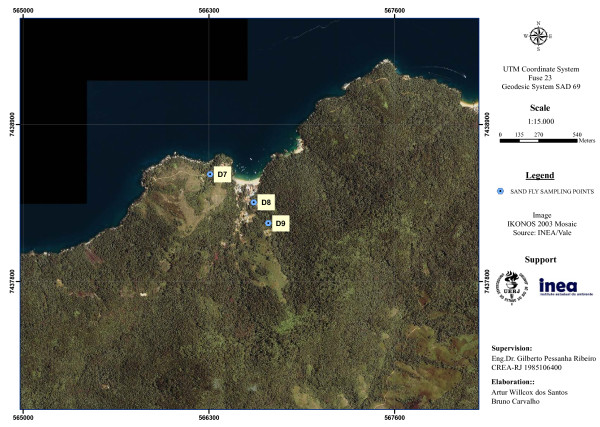
**Sand fly monitoring station 3, Praia Vermelha, showing sampled domiciles.** Ilha Grande, Angra dos Reis (RJ).

On each monitoring station, three domiciles were sampled (D1-D9) (Figures 2, 3, 4). The peridomestic area of sampled domiciles were very similar, having features which are likely to be suitable for sand fly breeding, such as dirt floor, fruitful trees and secondary forest. Some residences also had dogs, cats and chicken, and some dwellers commented that wild animals often appear near houses, e.g. opossums and rodents. Two additional sampling points were established at MS2 to capture sand flies for a *Leishmania* natural infection experiment (E1-E2) (Figure 3). Geographic coordinates of sampling points were obtained with a GPS receiver Garmin eTrex Vista HCx, in SAD69 Datum (Table 1).

**Table 1:** Geographic coordinates of sand fly sampling points

**Monitoring station**	**Sampling point**	**Latitude**	**Longitude**
MS1	D1	S 23°07’59.6”	W 44°10’11.6”
	D2	S 23°08’31.5”	W 44°10’17.0”
	D3	S 23°08’38.5”	W 44°10’02.0”
MS2	D4	S 23°07’04.7”	W 44°12’20.0”
	D5	S 23°07’01.5”	W 44°12’15.0”
	D6	S 23°06’11.9”	W 44°12’53.7”
	E1	S 23°06’55.5”	W 44°12’18.0”
	E2	S 23°06’51.1”	W 44°12’18.0”
MS3	D7	S 23°09’38.0”	W 44°21’08.0”
	D8	S 23°09’44.3”	W 44°20’57.2”
	D9	S 23°09’49.1”	W 44°20’53.6”

MS1: Vila do Abraão; MS2: Enseada das Estrelas; MS3: Praia Vermelha.

By looking at the three satellite images on the same scale (Figures 2, 3, 4), it is clear that MS1 has the largest deforested area. Vila do Abraão is known as the capital of the island and concentrates most tourism-related establishments (e.g. hotels, restaurants, stores and boat trip agencies). It is where the majority of tourists arrive at the island. The increase in tourism activities has contributed to a disorganized population growth with lack of proper infrastructure [19]. Comparatively, MS2 and MS3 are smaller fisherman villages, with less deforested area.

Captures were conducted monthly between July 2010 and September 2011. On each sampled domicile, three HP light traps [21] were installed: inside the domicile, in the peridomicile and in the nearest forest. The traps were exposed from 17:00 to 08:00, through four consecutive nights each month. Collected sand flies were sent to the laboratory for clarification, mounting in slides and species identification according to Young & Duncan [22].

In order to compare species abundance in the three monitoring stations, the Index of Species Abundance (ISA) [23] was calculated. This index aggregates information on relative abundance, as well as spatial distribution of collected individuals. The output values are standardized between 0 and 1 (SISA), so higher values mean the species is more abundant.

### ***Leishmania *** **natural infection**

Sand flies captured in MS2 (E1 and E2) and MS3 (D9) were sent to the laboratory for a *Leishmania* natural infection survey (58 unfed females and 57 males for contamination control). From each female, the two last abdominal segments were dissected in order to observe the internal genitalia and determinate its species according to Young & Duncan [22]. Since male species can be determined by observation of the external genitalia, no dissection was needed. The insects were then stored individually in 1.5 ml tubes at -18°C until DNA extraction and further analyzes.

DNA was extracted as previously described [24]. Multiplex Polymerase Chain Reaction (PCR) was designed to simultaneously amplify the cacophony gene IVS6 region (which is used as an internal control for the polymerase enzyme activity and DNA extraction in *Lutzomyia* sand flies), and the conserved kinetoplast DNA minicircle region from *Leishmania* spp. The amplified products further underwent dot blot hybridization with a *Leishmania (Viannia)*-specific biotinylated probe [24].

Rigorous procedures were assumed in order to control potential contamination: i) individual male sand flies were included as negative control in the DNA extraction step and in the PCR step; ii) DNA of artificially infected females were included as positive control; iii) all instruments and working areas were decontaminated with diluted chloride solution and ultraviolet light.

## Results

During the period of study, 1093 sand flies were captured from 16 species (Table 2). Four potential CL vectors were detected: *L. (N.) intermedia*, *L. migonei*, *L. (N.) flaviscutellata* and *L. (P.) ayrozai*. The main VL vector was also captured, *L. (L.) longipalpis*.

**Table 2:** Sand fly abundance and richness in monitoring stations

**Species**	**MS1**			**MS2**			**MS3**			**Total**		
	**M**	**F**	**M + F**	**M**	**F**	**M + F**	**M**	**F**	**M + F**	**M**	**F**	**M + F**
*Brumptomyia cunhai*	3	0	3	17	12	29	4	2	6	24	14	38
*Brumptomyia nitzulescui*	6	2	8	2	6	8	3	2	5	11	10	21
*Brumptomyia* spp.	1	0	1	0	1	1	0	2	2	1	3	4
Lutzomyia (Lutzomyia) longipalpis*	0	0	0	2	1	3	0	0	0	2	1	3
*Lutzomyia (Sciopemyia) microps*	0	0	0	0	2	2	0	1	1	0	3	3
*Lutzomyia edwardsi*	1	6	7	14	49	63	1	6	7	16	61	77
Lutzomyia migonei*	4	3	7	13	75	88	186	87	273	203	165	368
*Lutzomyia tupynambai*	2	29	31	3	38	41	0	5	5	5	72	77
*Lutzomyia termitophila*	0	0	0	0	2	2	0	0	0	0	2	2
*Lutzomyia firmatoi*	0	7	7	8	19	27	1	0	1	9	26	35
*Lutzomyia rupicola*	1	0	1	27	41	68	4	15	19	32	56	88
*Lutzomyia pascalei*	9	11	20	29	23	52	1	2	3	39	36	75
*Lutzomyia pelloni*	0	0	0	5	20	25	0	0	0	5	20	25
Lutzomyia (Nyssomyia) flaviscutellata*	1	4	5	0	3	3	0	0	0	1	7	8
Lutzomyia (Nyssomyia) intermedia*	0	1	1	16	70	86	52	45	97	68	116	184
Lutzomyia (Psychodopygus) ayrozai*	0	0	0	0	2	2	0	0	0	0	2	2
*Lutzomyia (Micropygomyia) schreiberi*	6	14	20	14	42	56	0	2	2	20	58	78
*Lutzomyia* spp.	0	0	0	1	4	5	0	0	0	1	4	5
Abundance	34	77	111	151	410	561	252	169	421	437	656	1093
Richness		11			16			11			16	

Ilha Grande, July 2010 to September 2011.

MS1: Vila do Abraão; MS2: Enseada das Estrelas; MS3: Praia Vermelha; M: Males; F: Females*.*

*Potential leishmaniases vector species.

Besides having the largest number of captured specimens, Enseada das Estrelas (MS2) had the highest species richness (S = 16). In Vila do Abraão (MS1), 111 specimens were captured from 11 species. Praia Vermelha (MS3) also had 11 species detected, with 421 specimens captured. *Lutzomyia (N.) intermedia* and *L. migonei* were present in the three monitoring stations. *Lutzomyia (N.) flaviscutellata* was present in MS1 and Enseada das Estrelas (MS2); *L. (L.) longipalpis* and *L. (P.) ayrozai* were found only in MS2 (Table 2).

**Figure 5:**
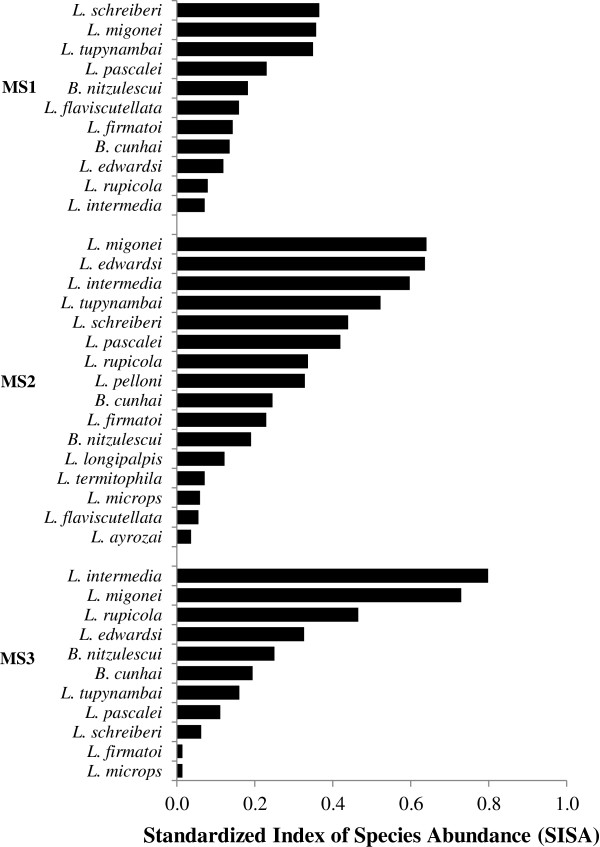
**Standardized Index of Species Abundance (SISA) of captured Phlebotomine sand flies in monitoring stations - MS1: Vila do Abraão; MS2: Enseada das Estrelas; MS3: Praia Vermelha.** Ilha Grande, Angra dos Reis (RJ), July 2010 to September 2011.

The most abundant species in MS1 were *L. (M.) schreiberi* (SISA = 0.37), *L. migonei* (SISA = 0.36) and *L. tupynambai* (SISA = 0.35). In MS2, *L. migonei* (SISA = 0.64) was the most abundant, followed by *L. edwardsi* (SISA = 0.64) and *L. (N.) intermedia* (0.60). MS3 had *L. (N.) intermedia* (SISA = 0.8), *L. migonei* (0.73) and *L. rupicola* (SISA = 0.47) as most abundant species (Figure 5).

The most common species captured inside houses and in peridomiciles were *L. migonei*, *L. (N.) intermedia* and *L. (M.) schreiberi*. The other potential leishmaniases vectors found, *L. (N.) flaviscutellata* and *L. (L.) longipalpis*, also occurred inside houses and in peridomiciles. *Lutzomyia (P.) ayrozai* occurred only inside the forest (Table 3).

**Table 3:** Sand fly abundance in domicile, peridomicile and forest

**Species**	**MS1**			**MS2**			**MS3**			**Total**		
	**D**	**P**	**F**	**D**	**P**	**F**	**D**	**P**	**F**	**D**	**P**	**F**
*Brumptomyia cunhai*	2	0	1	13	3	13	2	3	1	17	6	15
*Brumptomyia nitzulescui*	0	2	6	2	1	5	1	2	2	3	5	13
*Brumptomyia* spp.	0	1	0	1	0	0	1	0	1	2	1	1
Lutzomyia (Lutzomyia) longipalpis*	0	0	0	2	1	0	0	0	0	2	1	0
*Lutzomyia (Sciopemyia) microps*	0	0	0	0	0	2	0	1	0	0	1	2
*Lutzomyia edwardsi*	0	2	5	2	16	45	1	2	4	3	20	54
Lutzomyia migonei*	3	2	2	55	17	16	15	220	38	73	239	56
*Lutzomyia tupynambai*	0	4	27	1	8	32	0	4	1	1	16	60
*Lutzomyia termitophila*	0	0	0	1	0	1	0	0	0	1	0	1
*Lutzomyia firmatoi*	0	5	2	24	1	2	0	1	0	24	7	4
*Lutzomyia rupicola*	1	0	0	0	3	65	7	8	4	8	11	69
*Lutzomyia pascalei*	0	2	18	0	11	41	0	2	1	0	15	60
*Lutzomyia pelloni*	0	0	0	5	5	15	0	0	0	5	5	15
Lutzomyia (Nyssomyia) flaviscutellata*	0	3	2	0	0	3	0	0	0	0	3	5
Lutzomyia (Nyssomyia) intermedia*	0	1	0	29	49	8	31	33	33	60	83	41
Lutzomyia (Psychodopygus) ayrozai*	0	0	0	0	0	2	0	0	0	0	0	2
*Lutzomyia (Micropygomyia) schreiberi*	2	14	4	38	14	4	0	1	1	40	29	9
*Lutzomyia* spp.	0	0	0	1	2	2	0	0	0	1	2	2
Abundance	8	36	67	174	131	256	58	277	86	240	444	409

Ilha Grande, July 2010 to September 2011.

MS1: Vila do Abraão; MS2: Enseada das Estrelas; MS3: Praia Vermelha; D: Domicile; P: Peridomicile; F: Forest.

*Potential leishmaniases vector species.

**Figure 6:**
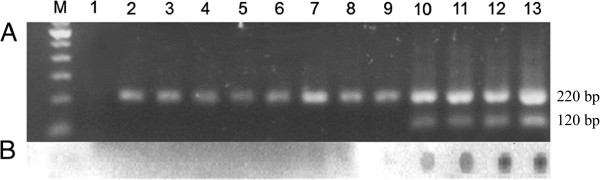
**Results of *Leishmania *natural infection survey. A)** 1.5% agarose gel electrophoresis of Multiplex PCR products. Lanes: M, molecular weight marker (100 bp); 1, negative control; 2-4, *Lutzomyia* sp. males; 5-9, negative females; 10-12, positive *Lutzomyia migonei* females; 13, positive control. **B)** Dot blot hybridization with *Leishmania (Viannia)*-specific biotinylated probe.

Three of 29 (10.3%) tested *L. migonei* females had positive results for natural infection with *Leishmania (Viannia)* sp. They were captured in peridomestic areas of D9, in MS3 (Table 4). The positive reactions for the PCR Multiplex and dot blot hybridization are shown in Figure 6.

**Table 4:** **Phlebotomine sand flies submitted to *Leishmania *** **natural infection survey**

**Species**	**Monitoring station**	**Positive**	**Negative**	**Total**	**Infection rate (%)**
*Lutzomyia edwardsi*	MS2	0	6	6	-
Lutzomyia migonei*	MS2	0	1	1	-
	MS3	3	26	29	10.3
*Lutzomyia tupynambai*	MS2	0	2	2	-
	MS3	0	1	1	-
*Lutzomyia rupicola*	MS2	0	13	13	-
	MS3	0	1	1	-
*Lutzomyia pelloni*	MS2	0	2	2	-
Lutzomyia (Nyssomyia) intermedia*	MS2	0	1	1	-
	MS3	0	2	2	-
Total		3	55	58	5.2

MS1: Vila do Abraão; MS2: Enseada das Estrelas; MS3: Praia Vermelha; M: Males; F: Females.

*Potential leishmaniases vector species.

## Discussion

The 16 sand fly species found in Ilha Grande corroborate with other studies performed in environmental protection areas of Atlantic Forest, which commonly detect about 10-30 sand fly species [25-27]. The observation of high species richness in forests and less impacted areas is common in the literature. In Colombia, Travi et al. [28] detected higher species richness in a forest reserve area when compared to captures made in a small village. Similar findings were shown in Brazil by Souza et al. [25], in Rio de Janeiro State, Alessi et al. [29] in São Paulo State and Pinto et al. [27] in Espírito Santo State. These studies also argue that, although species richness is lower, most vector species tend to be present in impacted areas. This is particularly evident in Praia Vermelha, locality with recent deforested areas by the landslides of 2010. The same vector species found in 1978 by Araújo Filho et al. [11,12] remain present in the deforested areas. Therefore, the occurrence of potential vectors *L. (N.) intermedia, L. migonei, L. (N.) flaviscutellata* and *L. (L.) longipalpis* in peridomestic areas of Ilha Grande’s villages must be highlighted.

Present results contribute with the first record of five species in Ilha Grande: *L. (Sciopemyia) microps*, *L. termitophila*, *L. firmatoi*, *L. rupicola* and *L. (Psychodopygus) ayrozai*. This was expected, because Ilha Grande has few published studies about its phlebotomine sand fly fauna, especially involving at least one year of monthly monitoring [14-16]. The exception is the study by Araújo Filho et al. [11,12], who performed sand fly captures in Praia Vermelha for 16 consecutive months. These five species had already been previously detected in Rio de Janeiro State [30] and in other Atlantic Forest areas of Espírito Santo State [27] and Paraná State [31].

The two most abundant species were *L. (N.) intermedia* and *L. migonei*, captured even inside houses, which was also observed in previous studies from Ilha Grande [11,12,14-16]. These species are considered important vectors of *Leishmania (Viannia) braziliensis* in Brazil’s Southeast region and are commonly captured in high abundance [24,32,33]. Other species found in high abundance, such as *L. edwardsi*, *L. tupynambai* and *L. rupicola*, are sylvatic species commonly found in Atlantic Forest areas [26,27,34-36]. *Lutzomyia tupynambai* was the most abundant sand fly species in forests of Serra da Tiririca State Park, Rio de Janeiro State, which suggests its common occurrence in this habitat [36]. These three species have no recorded evidence of importance in *Leishmania* transmission cycles. *Lutzomyia edwardsi* was found naturally infected by *Leishmania (V.) braziliensis* in São Paulo State, but it was not considered epidemiologically important especially because *L. migonei* was predominant in the studied locality [37].

*Lutzomyia (N.) intermedia*, captured in the three monitoring stations, is considered the main CL vector in Southeast Brazil, including Rio de Janeiro State [2,7]. It is commonly found as the most abundant sand fly species in Rio de Janeiro State [38-40] and in peridomestic areas of São Paulo and Espírito Santo States [2,41]. Its vectorial competence was already determined by findings of natural and experimental infection with *Leishmania (V.) braziliensis *[24,42-44].

The detection of *L. migonei* in high abundance also corroborates with previous studies of Atlantic Forest areas of Southeast Brazil, where it is usually found as the second-most abundant species and it is considered CL secondary vector [2,7]. It was first found naturally infected with flagellates by Pessoa & Coutinho [45] in São Paulo State. Afterwards, in Baturité, Ceará State, Azevedo et al. [46] also detected promastigotes in 0.2% of dissected *L. migonei* females, which were later identified as *Leishmania (V.) braziliensis *[47]. Pita-Pereira et al. [24], using the same molecular method as the present work, detected a 2% infection rate of *L. migonei* with *Leishmania (V.) braziliensis* in Jacarepaguá, Rio de Janeiro. In contrast with these low *Leishmania* natural infection rates, the high rate of *Leishmania (Viannia)* sp. infection detected in *L. migonei* in Praia Vermelha (10.3%) suggest the role of this sand fly species in CL transmission in this locality. These results complement the previous study of Araújo Filho et al. [11,12], who commented about *L. migonei* importance as CL vector in this locality.

It is also noteworthy that, during this study, the higher abundances of *L. migonei* were observed inside and next to chicken coops in Praia Vermelha, as previously observed by Araújo Filho et al. [11,12] in the same locality. The association between this species and chickens has been observed in other sand fly studies in Southeast Brazil [32,40].

Recently, *L. migonei* was found naturally infected with *Leishmania (L.) infantum chagasi* in São Vicente Férrer, Pernambuco State, suggested by the authors to be a potential VL vector in the locality [48]. Guimarães et al. [49] later detected, in the same locality, *L. migonei* naturally infected with *Leishmania (V.) braziliensis*. These findings demonstrate the capacity of *L. migonei* to maintain infection with two different species of *Leishmania*, although more studies are necessary to investigate the possibility of mixed infection and its role in VL transmission.

The occurrence of *L. (N.) flaviscutellata* in studied peridomestic areas is important, since this species is involved with the transmission of Diffuse Cutaneous Leishmaniasis (DCL), caused by *Leishmania (L.) amazonensis*. The low abundance observed can be an underestimate, since only light traps were used. This sand fly species is highly attracted to rodents [2,50], so animal-baited traps should be used for its capture, such as Disney traps [12,51]. Although *L. (N.) flaviscutellata* is predominantly distributed in the Amazon Forest, it was also found in Atlantic Forest areas of São Paulo State [52], Espírito Santo State [27], Bahia State [53] and Pernambuco State [54]. *Lutzomyia (N.) flaviscutellata* abundance and distribution in Rio de Janeiro State should be better studied, since the first DCL human case of the state was detected in 2007 in Paraty, a neighbor municipality of Angra dos Reis with very similar environmental characteristics to those from Ilha Grande [55].

*Lutzomyia (L.) longipalpis* was captured in Enseada das Estrelas, the only locality of Ilha Grande with a record of an autochthonous human case of VL. Considered as the main VL vector in Brazil, this species shows high adaptability to man-modified environments, especially because it has eclectic feeding habits and high anthropophily [1]. This sand fly is widely distributed in the country, occurring in especially high abundances in dry regions of Caatinga and Cerrado biomes, where it is frequently captured in peridomestic areas of rural and even urban regions [6,56]. However, in the present work, *L. (L.) longipalpis* was captured in a transition area between secondary forest and a recent deforested area. Its sylvatic origin has been discussed elsewhere [1]. This suggests that, in Ilha Grande, *L. (L.) longipalpis* is probably more common in forest areas, as it was also found in other Atlantic Forest areas of Rio de Janeiro, Espírito Santo and São Paulo States [36,57-60].

The detection of *L. (L.) longipalpis* was also important for the Health Surveillance of Angra dos Reis Municipality. Since the notification of the VL human case in 2005, it was still open in the Ministry of Health’s Disease Notification System, because the vector had not been captured. This finding, coupled with the detection of *Leishmania* infection in dogs from the locality [16], shows that a *Leishmania (L.) infantum chagasi* transmission cycle may be present and therefore surveillance must be constant.

*Lutzomyia (P.) ayrozai* was captured in low abundance inside the forest. This species is considered to be mainly sylvatic, and was already captured in Rio de Janeiro State in forest areas [25,26,61]. It is involved in the transmission of *Leishmania (V.) naiffi* in the Amazon, with rare human cases recorded. This rarity is discussed by Arias et al. [62] and Lainson et al. [63] as a consequence of the low attraction of *L. (P.) ayrozai* to man. In contrast, in areas of Atlantic Forest of southeast Brazil, this sand fly species was considered highly anthropophilic [61,64]. Since only two specimens of *L. (P.) ayrozai* were captured and there are no records of *Leishmania (V.) naiffi* outside north Brazil, this sand fly species probably doesn’t have a major role in leishmaniases transmission in Ilha Grande.

The present study demonstrates the occurrence of CL and VL vectors even after major environmental impacts (such as the landslides of 2010). This suggests that leishmaniases transmission profiles in the area may have shifted from essentially sylvatic to impacted and peridomestic areas, highlighting the need for constant epidemiologic and entomologic surveillance. In addition, Ilha Grande is an ecotourism area with tourists frequently trekking inside forests, where they may be exposed to vector contact. This can easily be reduced with use of insect repellents. People who live on the island need to have access to information about leishmaniases transmission, so that peridomestic cleaning can be carried out, to reduce establishment of vector breeding areas. Health education practices with the native population and information for tourists are suggested control actions that can be taken in Ilha Grande to reduce the burden of leishmaniases.

## Conclusions

Besides reinforcing the epidemiological importance of *L. (N.) intermedia* in Rio de Janeiro State, the role of *L. migonei* in CL transmission is highlighted with the finding of a high natural infection rate by *Leishmania (Viannia)* sp. The presence of *L. (N.) flaviscutellata* in peridomestic areas is of public health concern, since it is involved in the transmission of a rare and severe form of cutaneous leishmaniasis. The detection of *L. (L.) longipalpis* in the same locality where the only VL human case of Ilha Grande was recorded in 2005 allowed the closure of the case notification in the Ministry of Health’s Disease Notification System. This confirmed autochthonous transmission of visceral leishmaniasis in the island. Finally, the first detection of five sand fly species on the island demonstrates the importance of performing entomological surveys in Atlantic Forest areas. The occurrence of leishmaniases vectors in an area highly visited by tourists emphasizes the need for constant surveillance and health education practices.

## Abbreviations

CL: Cutaneous leishmaniasis; VL: Visceral leishmaniasis; FuSAR: Health Department of Angra dos Reis (Fundação Municipal de Saúde de Angra dos Reis); SINAN: Ministry of Health’s Disease Notification System (Sistema de Informação de Agravos de Notificação); ISA: Index of species abundance; SISA: Standardized index of species abundance; PCR: Polymerase chain reaction; MS: Sand fly monitoring station.

## Competing interests

The authors declare that they have no competing interests.

## Authors’ contributions

BMC conceived and designed the study, coordinated and participated in field work, analyzed data and wrote the manuscript; MM provided epidemiological and entomological data; WAC, ALFS and SMC participated in field work; TANCR performed the *Leishmania* natural infection survey; DPP coordinated the *Leishmania* natural infection survey; EFR coordinated the study, participating in its conception, design and discussion of results. All authors read and approved the final manuscript.
